# Magnetostatic Simulation and Design of Novel Radiofrequency Coils Based on Transverse Field Current Elements for Magnetic Resonance Applications

**DOI:** 10.3390/s24010237

**Published:** 2023-12-31

**Authors:** Giulio Giovannetti, Marcello Alecci, Angelo Galante

**Affiliations:** 1 Institute of Clinical Physiology, National Research Council (CNR), 56124 Pisa, Italy; giovannetti@ifc.cnr.it; 2 Department of Life, Health & Environmental Sciences (MESVA), University of L’Aquila, 67100 L’Aquila, Italy; marcello.alecci@univaq.it; 3 Gran Sasso National Laboratory, Istituto Nazionale di Fisica Nucleare, 67100 L’Aquila, Italy; 4 SPIN-CNR, c/o Department of Physical and Chemical Science, University of L’Aquila, 67100 L’Aquila, Italy

**Keywords:** magnetic resonance imaging, simulation, RF coils

## Abstract

Radiofrequency (RF) coils are key components in Magnetic Resonance (MR) systems and can be categorized into volume and surface coils according to their shapes. Volume RF coils can generate a uniform field in a large central sample’s region, while surface RF coils, usually smaller than volume coils, typically have a higher Signal-to-Noise Ratio (SNR) in a reduced Region Of Interest (ROI) close to the coil plane but a relatively poorer field homogeneity. Circular and square loops are the simplest and most used design for developing axial field surface RF coils. However, for specific MR applications, the use of dedicated transverse field RF coils can be necessary or advantageous. Building on a previously developed and validated RF coil simulator, based on the magnetostatic approach, here we explore the potential applications of novel multiple axial field and transverse field surface RF coils in non-standard configurations. We demonstrate via numerical simulations that simple volume RF coils, matching a Helmholtz-like design, can be built with two identical transverse field RF coils separated by a given distance. Following well-known principles, the SNR of such novel configurations can be improved by a factor of up to √2 by combining two 90^°^ rotated coils, producing, inside a central ROI, a circularly polarized B_1_ field.

## 1. Introduction

Radiofrequency (RF) coils are key components in Magnetic Resonance (MR) systems. For obtaining high-quality data, RF coils should be able to provide a wide Field-Of-View (FOV) with high magnetic field homogeneity for RF pulse transmission and to achieve a high Signal-to-Noise Ratio (SNR) for MR signal reception. While volume RF coils are generally used either for transmission only or transmission/reception, surface RF coils are mainly used as receivers due to the localized higher SNR, albeit with much lower RF field spatial homogeneity [1,2].

Classical surface RF coils are constituted by square or circular electrical conductor loops, which produce a B_1_ field perpendicular to the RF coil plane in the Region-Of-Interest (ROI) placed around the coil axis, whose amplitude decreases along it. 

Differently shaped surface RF coils have been designed over the years for improving the MR SNR performance or the ROI localization, even exploiting the B_1_ field parallel to the coil plane [3]. Although few works in the literature are focused on exploiting the transverse components of the B_1_ field of loop RF coils [4,5], more are dedicated to different surface coil topologies, in particular the so-called Butterfly Coil (BC) and Figure-of-eight Coil (FC). Their design has two central straight current elements producing a B_1_ field that, in the ROI located close to the RF coil axis, has a direction orthogonal to that axis and parallel to the coil plane [6]. 

In a previous paper [7], authors reviewed the literature regarding BC and FC RF coils devoted to MRI proton applications (from 0.25 to 7 T), parallel MRI, and non-proton MRI/MRS (^13^C, ^23^Na, ^31^P). In the paper, a Biot–Savart law-based simulator was developed for the RF magnetic field pattern estimation of a standard RF Square Loop Coil (SLC), Square BC (SBC), and Square FC (SFC), whose accuracy was validated through workbench tests performed at 100 MHz on prototypes of the three different configurations [7]. The simulation code was freely provided to let other researchers optimize further RF coil designs accordingly to their specific application. Moreover, two novel RF coil structures constituted by, respectively, two overlapped orthogonal SBCs and SFCs were also simulated.

It is important to note that magnetic field modelling with a magnetostatic approach can be used for optimizing the performance of coils for different applications, not only for MRI, by taking advantage of computer simulations. The literature reports examples of different systems, comprising pickup coils employed in vibrating sample magnetometers [8], whose geometries and arrangements strongly influence the measurement sensitivity, or circular and DD-type coils designed for maximizing the transmission process efficiency in wireless power transmission applications [9]. The Biot–Savart law was also applied for the magnetic field distribution estimation of Helmholtz coils used for archaeomagnetic measurements on fired archaeological materials and volcanic products, with the goal to design homogeneous magnetic field inductors [10].

In the present work, based on a previously developed and validated code [7], we propose novel multiple axial field and transverse field surface RF coils in non-standard configurations. This paper extends the simulations to different quadrature surface coils constituted by, respectively, a combination of SLC-SBC, SLC-SFC, and SFC-SBC, useful for improving by a factor of up to √2 the SNR provided by a single surface RF coil and for producing a circularly polarized B_1_ field in the central ROI with the field rotating in a plane orthogonal or parallel to the coils depending on the configuration. We also propose for the first time three novel volume RF coils based on pairs of surface coils. These configurations, named Helmholtz-like due to their similarity with the well-known Helmholtz pair design, were simulated by using two identical surface RF coils (SLC-SLC, SFC-SFC, and SBC-SBC) separated by a given distance.

The manuscript is organized as follows: after a brief description of the Biot–Savart law and the isolated SLC, SBC, and SFC RF coils, the quadrature structures are introduced and simulated. Successively, the Helmholtz-like volume RF prototypes are theoretically described and simulated. Finally, a discussion and conclusions follow.

## 2. Materials and Methods

### 2.1. Magnetic Field Pattern Calculation

The calculation of the RF coils’ magnetic field pattern was performed with the Biot–Savart law numerical integration, able to estimate the free space *B*_1_ field distribution produced by an electric current *I* which flows in an arbitrary closed contour *C* as [1]

(1) $$B\left(r\right)=\frac{\mu_{0}I}{4\pi }\int_{C}\frac{dl\wedge R}{R^{3}}$$

where *μ*_0_ = 4*π* × 10^−^^7^ Henry per meter (H/m) is the free space permeability, *dl* is the infinitesimal vector tangential to *C*, and *R* is the distance between the conductor path and observation point. As from Equation (1), the calculation of the magnetic field generated by the currents along the RF coil conductors can be performed by subdividing the coil path into segments, calculating each segment’s magnetic field contribution, and summing their contribution to obtain the total magnetic field.

For the RF coil simulations, the Biot–Savart law was implemented in IDL 6.0 (Interactive Data Language, Visual Information Solutions, Boulder, CO, USA) and its use implies a nearly static field assumption that holds only when the coil dimensions are much lower than the wavelength. In the simulations, the conductor width was neglected with respect to the wavelength, and the magnetic field generated by the currents was evaluated assuming the thin wires’ approximation [11].

The magnetic field patterns calculated with Equation (1), shown in the following sections, both refer to the transmit (B_1_^+^) and receive (B_1_^−^) fields, which according to the reciprocity theorem [12] coincide with the magnetostatic approach.

### 2.2. The Isolated RF Coils

**Figure 1:**
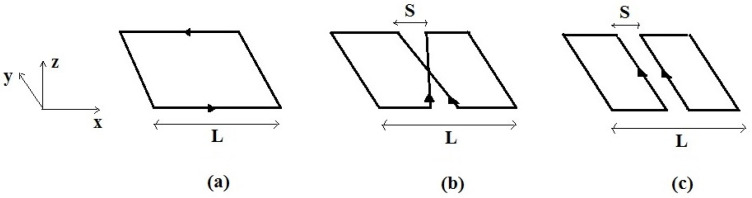
The single RF coils: (**a**) SLC; (**b**) SBC; (**c**) SFC. *L* is the overall size and *S* is the separation between linear current elements.

Figure 1 shows the single RF coil models simulated: the SLC (side *L*), the SBC (side *L*, spacing *S*), characterized by two linear current elements crossing at the coil center, and the SFC (side *L*, spacing *S*), in which two linear and parallel currents flow in the same direction.

With respect to the coils’ plane (*x*-*y*), the SLC generates, along its axis, an axial field with *B*_1_ directed along *z*. SBC and SFC coils belong to a different class since along their axis they generate a *B*_1_ field directed along *x* (Figure 1). The magnetostatic simulation results of these RF coils, as well as workbench tests performed at 100 MHz, are reported in a previous paper [7]. 

### 2.3. The Quadrature Configurations

Quadrature RF coils generate mutually orthogonal *B*_1_ fields creating a circular polarization within an ROI, thus allowing a quadrature operation which halves the RF drive power over the linear excitation in transmit mode and, by the reciprocity theorem [12], increases the SNR by a maximum factor of √2 in the receive mode [1,2,13].

For temporomandibular joint imaging, Hyde et al. [14] proposed a quadrature detection surface coil, constituted by a planar pair and counter-rotating current (CRC) loop-gap resonators, providing an SNR gain of 2.5 and a scanning time reduction by a factor of two respective to the single coils. Another quadrature surface coil configuration, constituted by a BC and a circular loop, was employed for human heart imaging at 7 T, with the coil geometry optimized for achieving coverage across the entire heart [15].

**Figure 2:**
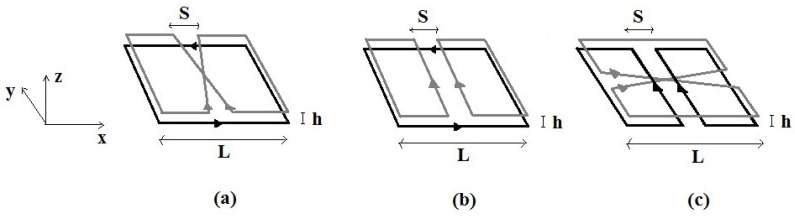
The quadrature surface RF coils constituted by the combinations of: (**a**) SLC-SBC; (**b**) SLC-SFC; (**c**) SFC-SBC. *L* is the overall size, *S* the separation between elements, and *h* the distance between the coil planes.

In this paper, three different quadrature surface RF coil configurations are proposed and simulated, namely: (i) SLC-SBC (Figure 2a), (ii) SLC-SFC (Figure 2b), and (iii) SFC-SBC (Figure 2c). The simulations of the RF magnetic field pattern were performed using a relatively small distance *h* between the RF coil planes (Figure 2).

Due to coils’ symmetry considerations and assuming perfect geometries, these configurations present no inductive coupling among the RF coils and can, in principle, be realized without the need for specially designed decoupling circuital elements. Should the coupling be not negligible, it can be further minimized by choosing different lateral sizes *L* for the two RF coils to reduce the current elements in proximity, hence minimizing the electrical coupling. Without loss of generality, we will consider in the following two same-size RF coils: when the above discussed size adjustment is necessary it will have little impact on the RF field profile close to the *z*-axis. 

The proposed quadrature RF coils can be employed for both transmitting and receiving quadrature fields. Often, an ideal MRI setup comprises a transmit-only volume coil for achieving a uniform excitation in a large volume and one or multiple receive-only coils with high local sensitivity. The proposed quadrature RF configurations can be adapted to this scheme once decoupling circuits are inserted for minimizing the interaction between the transmit and receive coils [16].

### 2.4. The Helmholtz-like Configurations

The Helmholtz RF coil geometry is the simplest example of an arrangement of two parallel surface RF coils producing, in the central region, a homogeneous magnetic field with a relatively high SNR. In general, such design is constituted by two identical and coaxial circular loops with in-phase currents flowing in each loop, and the loop radiuses are equal to the distance between the two loops [1]. In this configuration, a highly uniform B_1_ field along the RF coils axis is produced, where the prime and second derivative of the magnetic field along the coils’ axis is zero in the central point. A transmit/receive Helmholtz coil was designed for MRI/MRS studies in small phantoms (constituted by metabolic compounds part of the brain) with a clinical 3T scanner [17], while a Helmholtz coil paired with a V-shape structure was designed and built for the imaging of dogs in a V-shape permanent MRI system [18]. In both works, axial surface coils were employed, while we suggest a similar configuration to be realized with two transverse RF coils. The main difference is that for transverse coils, to add the contributions of the central current elements, the currents should flow with opposite phases. Considering the symmetrical properties of the resultant RF magnetic field in the space between the coils, we will call them Helmholtz-like and propose them as a new way to combine surface RF coils to image a larger sample’s volume. The transverse RF coils are used to reach a high SNR close to the coil’s surface and we expect their Helmholtz-like configurations to be useful when the inter-coil distance is small (i.e., of the order of 2*S*). We believe that this can achieve applications in human fingers, small animal arts, or ex vivo slab specimens. 

**Figure 3:**
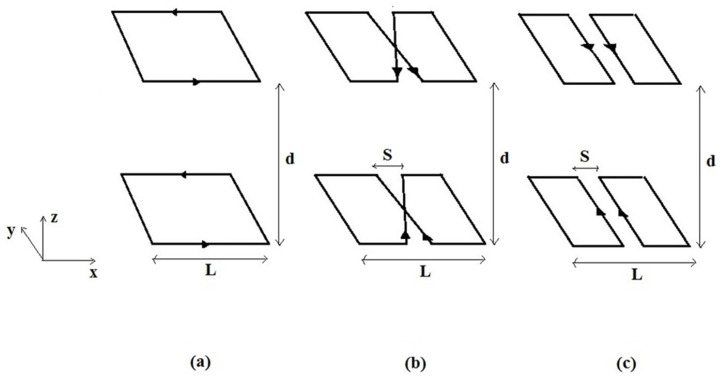
The Helmholtz-like RF coils constituted by two (**a**) SLCs; (**b**) SBCs; and (**c**) SFCs. *L* is the overall size, *S* the separation between elements, and *d* the distance between the RF coil planes.

In this work, we consider three Helmholtz-like coil configurations, constituted by a couple of SLCs (Figure 3a), SBCs (Figure 3b), and SFCs (Figure 3c), each one having a lateral size *L*, a separation between elements *S*, and a distance between the RF coil planes *d*.

The above configurations can present the drawback of relevant coupling among the two resonant RF coils. This coupling introduces a frequency splitting for the eigenmodes described by co-rotating and anti-rotating currents in the two coils. Even if it is possible to tune the RF coils to have the desired mode (co-rotating for configuration 3a, counter-rotating for configurations 3b and 3c) at the Larmor frequency, this solution is suboptimal since the correlation among the couple of RF coils includes the noise component, thus limiting the expected SNR gain of the quadrature operation [19]. An avenue to practically adopt each of the configurations in Figure 3 is to decouple the two resonant RF coils by introducing an extra decoupling capacitor in between them [20]. This can be effective but introduces some extra complexity in the practical realization.

Another possibility for the SFC and SBC Helmholtz pairs is to rotate, along the z axis, the upper coil by 90° with respect to the lower one. These configurations, denoted as quadrature Helmholtz pairs in the following, are advantageous for two reasons. First, we obtain two orthogonal components of the RF magnetic field producing a circularly polarized *B*_1_ field in the *x*-*y* transverse plane. Second, the 90° rotation minimizes the mutual coupling among the two RF coils, and assuming perfect geometry, the inductive coupling is reduced to zero, with only a much smaller residual electrical coupling left. This significantly reduces the frequency splitting of the resonant frequency, as compared to the frequencies of the two identical isolated RF coils, thus avoiding the inclusion of extra elements for decoupling them.

### 2.5. Calculation of the Optimal Helmholtz-like Configuration

**Figure 4:**
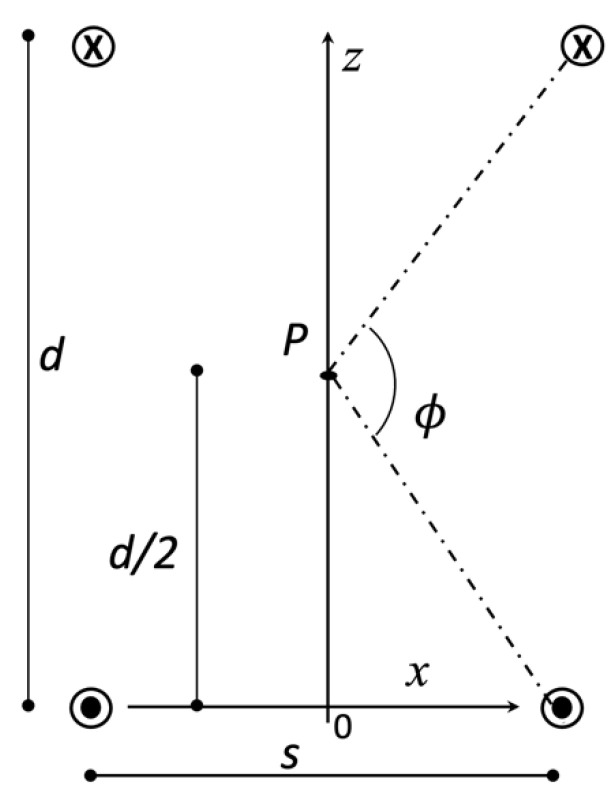
Two parallel SFC RF coils in a Helmholtz-like configuration in a lateral section view. Four straight and parallel current patterns are visible: two in the lower part and two in the upper one.

In the following, we will start with the calculation of the optimal configuration for two SFC coils (Figure 3c) disposed on parallel planes with a mutual distance d, with parallel central current elements but currents flowing in opposite directions (see Figure 4). In our simplified approach, we will neglect the contribution of the return current paths, which are at a much larger distance from the central ROI than the central straight and parallel current elements. The magnetic field of a single straight current segment in any point P belonging to the plane orthogonal to the current segment and passing its central point can be written as $\frac{\mu_{0}I}{2\pi }\frac{\sin\theta }{h_{1}}\;,$ where $h_{1}$ is the distance of P from the segment and *θ* is the angle formed by the current segment and the segment connecting any of its extremities with P. Let us consider the RF magnetic field produced by a pair of identical and parallel straight current elements, located at distance *S*. If we choose a point P along the *z* axis (see Figure 4) and calculate the *x* component of the magnetic field, we obtain

(2) $$B_{2,x}\left(z\right)=A\frac{z}{z^{2}+{\left(S/2\right)}^{2}}\;$$

with $A=\left(\mu_{0}I/\pi \right)\sin\theta$. In the Helmholtz-like configuration of two SFCs with central current elements parallel but with opposite currents, the magnetic field along the *z* axis can be written as

(3) $$B_{4,x}\left(z\right)=B_{2,x}\left(z\right)+B_{2,x}\left(d-z\right)\;.$$

Since ${𝜕}_{z}B_{4,x}\left(z\right)={𝜕}_{z}B_{2,x}\left(z\right)-{𝜕}_{z}B_{2,x}\left(d-z\right)\;$, it is easy to verify that ${𝜕}_{z}B_{4,x}\left(z=d/2\right)=0$, as expected from the symmetry of the current sources. Halfway between the two coils, the magnetic field’s first derivative is zero, and its local variations are minimized. We now search for the configuration that realizes the most homogeneous RF magnetic field along the *z*-axis, looking for solutions that satisfy the extra constraint ${𝜕}_{z}^{2}B_{4,x}\left(z=d/2\right)=0$. The latter condition can be fulfilled because we still have a free parameter: the non-dimensional ratio d/s. Since ${𝜕}_{z}^{2}B_{4,x}\left(z\right)={𝜕}_{z}^{2}B_{2,x}\left(z\right)+{𝜕}_{z}^{2}B_{2,x}\left(d-z\right)$, we have that ${𝜕}_{z}^{2}B_{4,x}\left(d/2\right)=2{𝜕}_{z}^{2}B_{2,x}\left(d/2\right)$ and our constraint reduces to ${𝜕}_{z}^{2}B_{2,x}\left(d/2\right)=0$. A direct calculation from Equation (2) shows that the constraint is fulfilled for $d/s=\sqrt{3}$, which corresponds to $\phi =120^{{}^{\circ}}$ (see Figure 4). This result is not surprising since the current distribution in Figure 4 is analogous to a saddle RF coil in a section view orthogonal to the axis of the cylinder that hosts the conductor. The two currents, on the left and right side of the *z*-axis in Figure 4, correspond to the two windings of the saddle coil and it is well-known that its optimal configuration, nulling all magnetic field’s second derivatives at the center, is realized for $\phi =120^{{}^{\circ}}$ and a given height-to-diameter ratio [21]. 

If we consider a similar configuration but rotate the upper SFC coil by $90^{{}^{\circ}}$ along the *z*-axis, we obtain two orthogonal components of the RF magnetic field. In the *x*-*y* plane, the transverse field amplitude $B_{4,xy}\left(z\right)$ is equal to

(4) $$B_{4,xy}\left(z\right)=\sqrt{B_{2,x}^{2}\left(z\right)+B_{2,y}^{2}\left(d-z\right)}\;.$$

Considering the derivatives along *z*, we obtain the following two equations:

(5) $${\dot{B}}_{4}\left(z\right)\propto {\left[B_{2}^{2}\left(z\right)+B_{2}^{2}\left(d-z\right)\right]}^{-\frac{1}{2}}\cdot \left(B_{2}\left(z\right){\dot{B}}_{2}\left(z\right)-B_{2}\left(d-z\right){\dot{B}}_{2}\left(d-z\right)\right)$$

(6) $${\ddot{B}}_{4}\left(z\right)\propto {\left[B_{2}^{2}\left(z\right)+B_{2}^{2}\left(d-z\right)\right]}^{-\frac{3}{2}}\cdot {\left(B_{2}\left(z\right){\dot{B}}_{2}\left(z\right)-B_{2}\left(d-z\right){\dot{B}}_{2}\left(d-z\right)\right)}^{2}+2{\left[B_{2}^{2}\left(z\right)+B_{2}^{2}\left(d-z\right)\right]}^{-\frac{1}{2}}\;\cdot \left({\dot{B}}_{2}\left(z\right){\dot{B}}_{2}\left(z\right)+B_{2}\left(z\right){\ddot{B}}_{2}\left(z\right)+{\dot{B}}_{2}\left(d-z\right){\dot{B}}_{2}\left(d-z\right)+B_{2}\left(d-z\right){\ddot{B}}_{2}\left(d-z\right)\right)$$

where, for brevity, we used the point formalism for spatial derivatives and omitted the component for the field generated by the two coils. The latter is justified since the $B_{2,x}$ component of the magnetic field generated by the lower *z*-axis RF coil has the same *z* dependence of the $B_{2,y}$ field generated by the upper *z*-axis one. From Equation (5), it is evident that ${\dot{B}}_{4}\left(z=d/2\right)=0$ and the same holds for the first term in Equation (6). From Equation (2), it can be demonstrated that for $d/s=\sqrt{3}$, the term ${\dot{B}}_{2}\left(z=d/2\right)$ is different from zero, while ${\ddot{B}}_{2}\left(z=d/2\right)=0$, thus leaving the second term in Equation (6) different from zero. We conclude that in the central point of the *z*-axis it is not possible any more to null both the first and second derivative: the advantages of circular polarization and reduced coupling among the resonant circuits arise at the expenses of a reduced field uniformity along the *z*-axis. 

For the SBC configuration, we can proceed in a similar way. For a single SBC RF coil, with the central elements forming a small angle γ with respect to the *y*-axis ($\gamma =\tan^{-1}S/L$), we obtain

(7) $$B_{2,x}\left(z\right)=A\frac{\sin\gamma }{z}$$

Let us now consider the magnetic field produced along the *z*-axis, passing the central elements’ crossing point, for two parallel SBC coils having the same orientation in the *x*-*y* plane, but at distance *d* along the *z* axis. We can use the above formula together with Equation (3) and prove that ${\dot{B}}_{4}\left(z\right)$ is zero for z=d/2, although it is impossible to null the second derivative adjusting the free parameter (γ) as we did before for d/s. The reason is that while for the SFC coil the term ${\ddot{B}}_{2}\left(z\right)$ changes its sign, for the SBC this does not happen. It turns out that for SBC, ${\ddot{B}}_{4}\left(z\right)={\ddot{B}}_{2}\left(z\right)+{\ddot{B}}_{2}\left(d-z\right)$ is the sum of two non-zero terms with the same sign and it is never zero for *z* > 0. We conclude that for SBC coils in a Helmholtz-like configuration, the optimal configuration is realized for z=d/2, but only the first derivative is nulled, not the second, and a more restricted homogeneity region along the *z*-axis can be expected with respect to the SFC case. As previously discussed for the SFC case, the upper SBC coil can also be $\mathrm{rotated}\;90^{{}^{\circ}}$ along the *z*-axis to obtain a more efficient circular polarization magnetic field and null the inductive coupling among the two RF coils. In this case, the argument runs like the SFC case, and again we can only null the first derivative for z=d/2.

## 3. Results

### 3.1. The Quadrature Configurations

**Figure 5:**
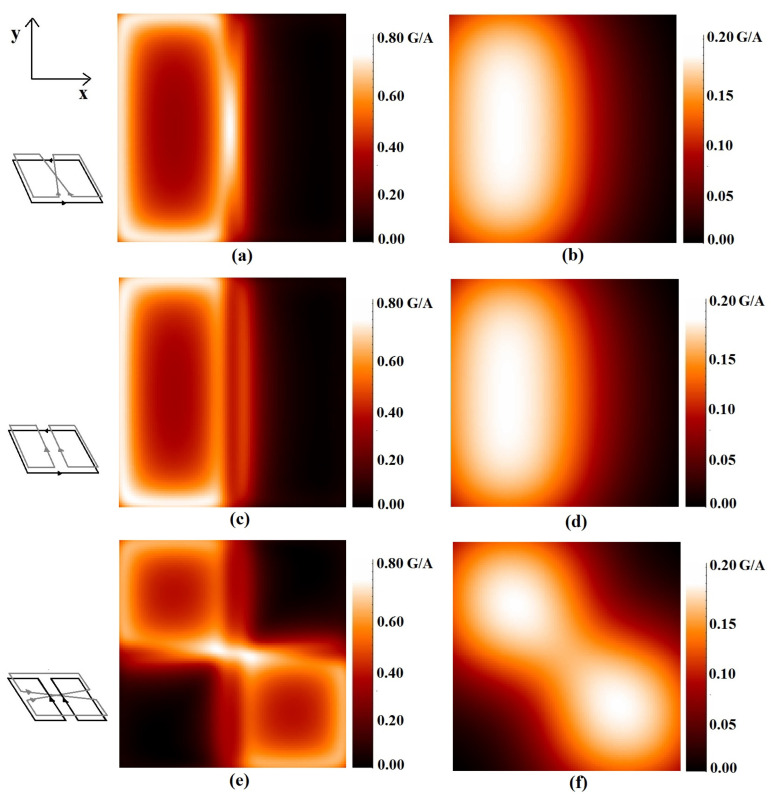
Distributions of the magnetic field modulus (Gauss/A), in the magnetostatic approximation, for different quadrature configurations (*L* = 10 cm, *S* = 1 cm, and *h* = 0.8 mm): (**a**,**b**) SLC-SBC; (**c**,**d**) SLC-SBC; (**e**,**f**) SLC-SFC. The first column refers to the *z* = 0.6 cm plane, and the second to the *z* = 3 cm plane.

Figure 5 depicts the magnetic field modulus calculated in the *x*-*y* plane of the three quadrature coil configurations of Figure 2 (*L* = 10 cm, *S* = 1 cm, and *h* = 0.8 mm), which produce orthogonal *B*_1_ fields in a restricted region along the coils’ axis. Specifically, Figure 5 shows the magnetic field calculated at *z* = 0.6 cm and *z* = 3 cm for the SLC-SBC (Figure 5a,b), SLC-SFC (Figure 5c,d), and SFC-SLC (Figure 5e,f) RF coils. 

Figure 5a,c,e show that for *z* = 0.6 cm, the *B*_1_ profiles are quite different. In particular, the SLC-SBC *B*_1_ configuration (Figure 5a) in the central area has a maximum field intensity close to the coil axis due to the SBC central current lines crossing. The pattern of the SLC-SFC configuration (Figure 5b) is different from the previous one, with a reduced field intensity along the *z* axis. Finally, the SFC-SBC configuration (Figure 5c) has a diagonally elongated hot spot in the central area.

The third column in Figure 5b,d,f shows the same coils’ configurations but at a larger distance (*z* = 3 cm). When *z* > *S*, the differences between the SFC and SBC profiles are small and the SLC-SFC and SLC-SBC field profiles become similar, while the SFC-SBC pattern preserves its peculiar diagonal symmetry. 

**Figure 6:**
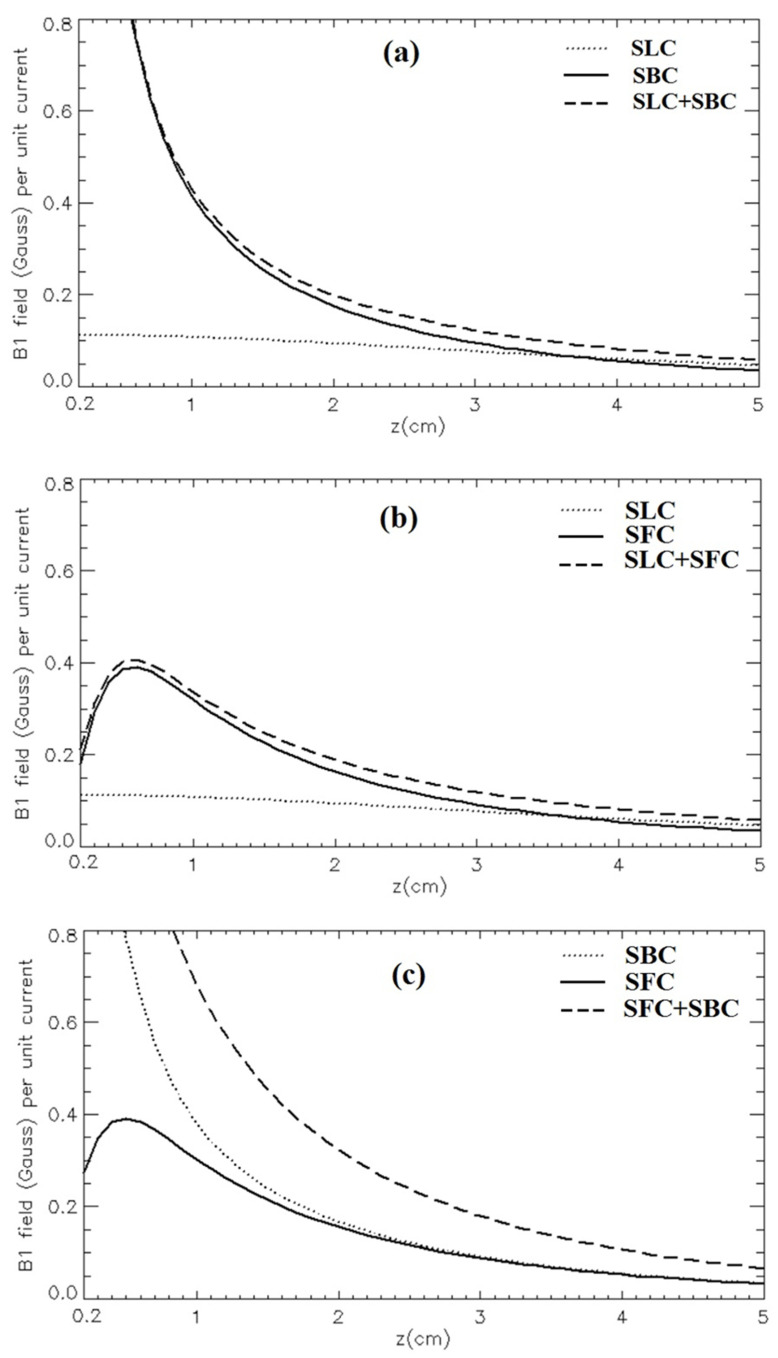
Simulated magnetic field profiles (Gauss/A) along the *z*-axis for the quadrature configurations, compared with the single coil case: (**a**) SLC-SBC; (**b**) SLC-SFC; (**c**) SFC-SBC.

Figure 6 depicts the magnetic field patterns along the RF coils’ axis for the three quadrature configurations compared to the ones provided by the single coil. All plots show the √2 magnetic field gain expected by the quadrature configuration. For a fair comparison among different configurations, and to keep visibility of the magnetic profiles for configurations including SLC and SFC coils, we fixed the vertical scale of all plots to the 0–0.8 Gauss range. The z→0 diverging behaviour of configurations with the SBC coil is not fully described in the plots but in most applications the samples are located at least several mm away from the coils to avoid electric field hot spots. 

As shown in Figure 6a, the SLC-SBC field pattern decreases rapidly with *z*, but at *z* = 2 cm the magnetic field amplitude is over two times with respect to the single SLC. The SLC-SFC configuration (Figure 6b) shows a field pattern that along the *z*-axis is zero at *z* = 0, reaches its maximum at about *z* = 0.5 cm, and then decreases less rapidly than the single SFC. Finally, the SFC-SBC field distribution, shown in Figure 6c, decreases rapidly with *z*, and at *z* = 2 cm, the field amplitude is about two times with respect to the single SFC.

### 3.2. The Helmholtz-like Configurations

**Figure 7:**
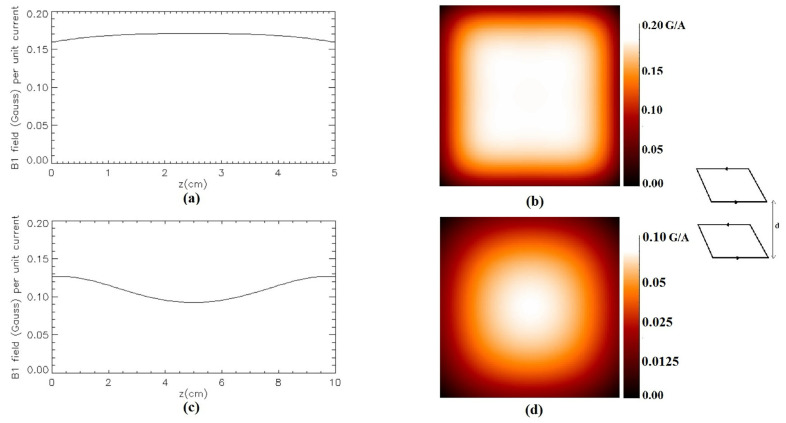
Simulated magnetic field (Gauss/A) for the Helmholtz-like configuration constituted by two parallel SLCs: (**a**) along the *z*-axis for *d* = 5 cm; (**b**) in the *x*-*y* plane at *z* = 2.5 cm for *d* = 5 cm; (**c**) along the *z*-axis for *d* = 10 cm; (**d**) in the *x*-*y* plane at *z* = 5 cm for *d* = 10 cm.

Figure 7 shows the magnetic field modulus of the Helmholtz-like RF coil configuration constituted by a couple of SLCs (*L* = 10 cm) for two different distances between the loops. Figure 7a,c show, respectively, the magnetic field profile plot along the *z*-axis for a coil structure with *d* = 5 cm and *d* = 10 cm, while Figure 7b,d depict, respectively, the field distribution in the *x*-*y* plane located half-way between the coils (*z* = 2.5 cm for *d* = 5 cm and *z* = 5 cm for *d* = 10 cm).

**Figure 8:**
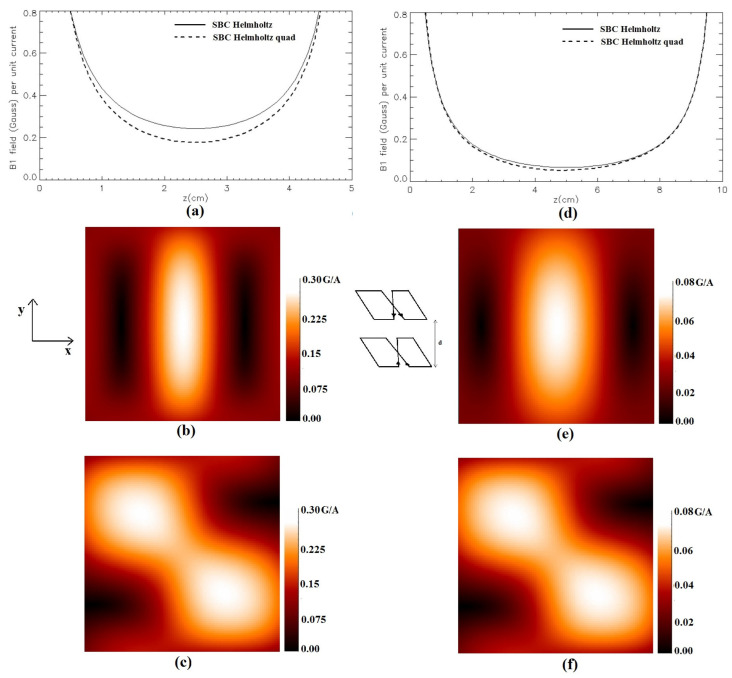
Simulated magnetic field (Gauss/A) for the Helmholtz-like configuration constituted by two parallel SBCs: (**a**) along the *z*-axis for *d* = 5 cm; (**b**) in the *x*-*y* plane at *z* = 2.5 cm for *d* = 5 cm; (**c**) in the *x*-*y* plane at *z* = 2.5 cm for *d* = 5 cm for quadrature SBC; (**d**) along the *z*-axis for *d* = 10 cm; (**e**) in the *x*-*y* plane at *z* = 5 cm for *d* = 10 cm; (**f**) in the *x*-*y* plane at *z* = 5 cm for *d* = 10 cm for quadrature SBC.

Figure 8 depicts the modulus of the magnetic field of the Helmholtz-like configuration constituted by a couple of SBCs (*L* = 10 cm, *S* = 1 cm) for two values of the coils’ distance. Figure 8a,d show, respectively, the magnetic field profile plot along the *z*-axis for a coil structure with *d* = 5 cm and *d* = 10 cm. As in Figure 6, we used the vertical scales excluding the first mm close to the coils’ planes. Figure 8b,e depict, respectively, the field distribution in the *x*-*y* plane located half-way between the coils (*z* = 2.5 cm for *d* = 5 cm and *z* = 5 cm for *d* = 10 cm). Finally, Figure 8c,f show, respectively, the field distribution in the *x*-*y* plane located half-way between the coils (*z* = 2.5 cm for *d* = 5 cm and *z* = 5 cm for *d* = 10 cm) for the quadrature SBC.

**Figure 9:**
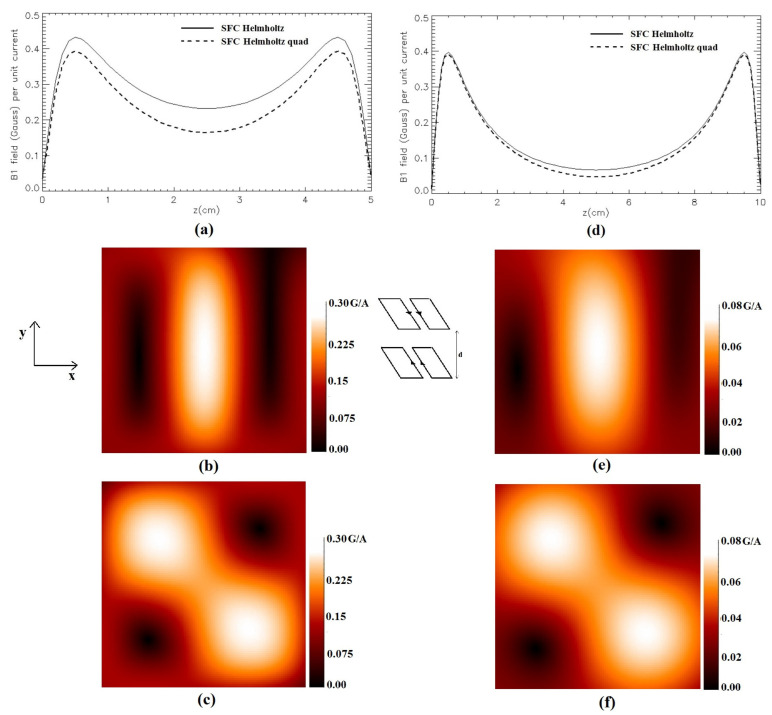
Simulated magnetic field (Gauss/A) for a Helmholtz-like configuration constituted by two parallel SFCs: (**a**) along the *z*-axis for *d* = 5 cm; (**b**) in the *x*-*y* plane at *z* = 2.5 cm for *d* = 5 cm; (**c**) in the *x*-*y* plane at *z* = 2.5 cm for *d* = 5 cm for quadrature SLC; (**d**) along the *z*-axis for *d* = 10 cm; (**e**) in the *x*-*y* plane at *z* = 5 cm for *d* = 10 cm; (**f**) in the *x*-*y* plane at *z* = 5 cm for *d* = 10 cm for quadrature SLC.

In Figure 9, the magnetic field modulus of the Helmholtz-like coil configuration constituted by two SFCs (*L* = 10 cm, *S* = 1 cm) for different distances between the coils is shown. Figure 9a,c show, respectively, the magnetic field profile plot along the *z*-axis for a coil structure with *d* = 5 cm and *d* = 10 cm, while Figure 9b,d depict, respectively, the field distribution in the *x*-*y* plane located half-way between the coils (*z* = 2.5 cm for *d* = 5 cm and *z* = 5 cm for *d* = 10 cm).

**Figure 10:**
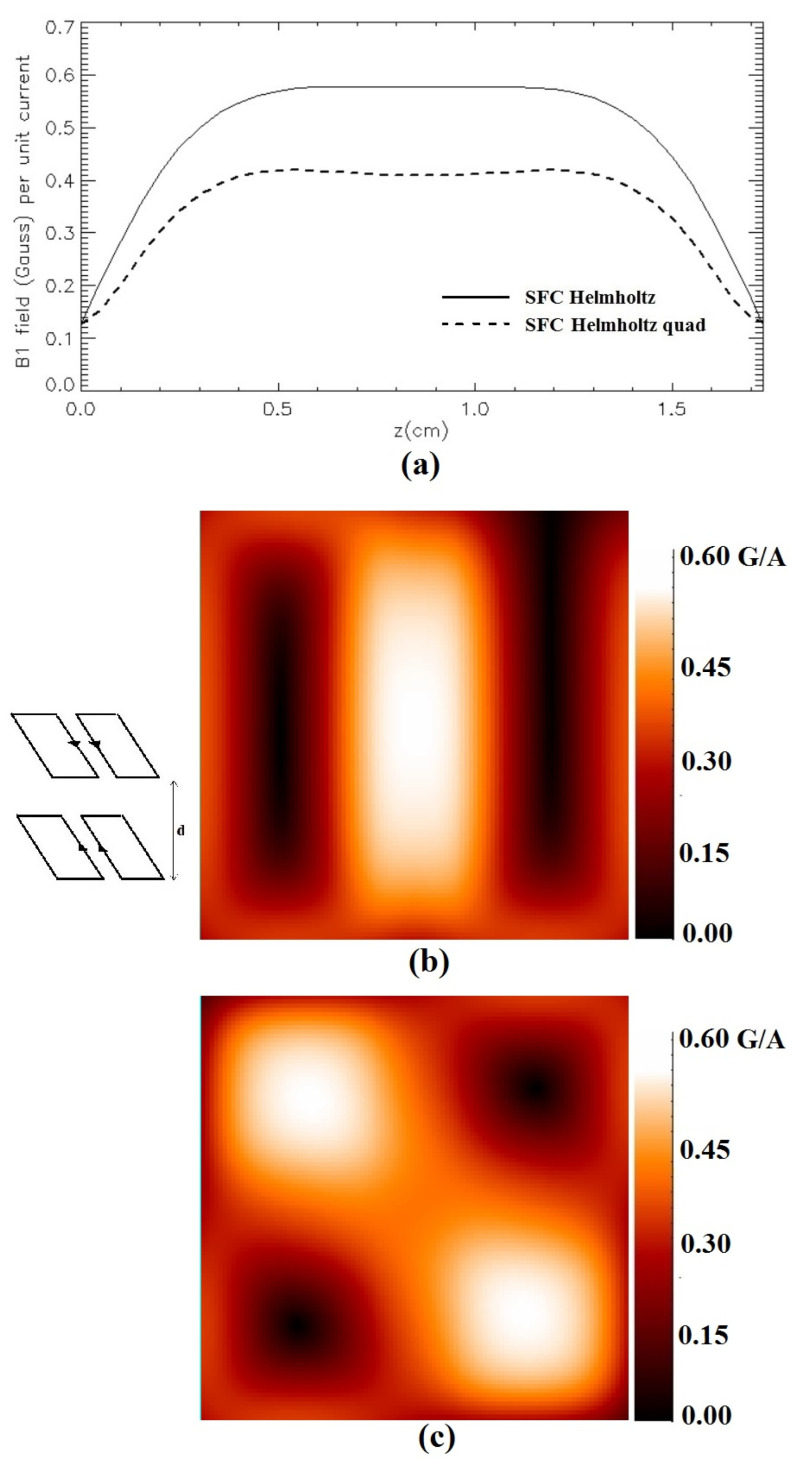
Simulated magnetic field (Gauss/A) for a Helmholtz-like configuration constituted by two parallel SFCs (*L* = 5 cm, *S* = 1 cm, *d* = √3 cm): (**a**) along the *z*-axis; (**b**) in the *x*-*y* plane at *z* = √3/2 cm; (**c**) in the *x*-*y* plane at *z* = √3/2 cm for quadrature SLC.

Figure 10 shows an example of the magnetic field modulus of the Helmholtz-like coil configuration constituted by two SFCs (*L* = 5 cm, *S* = 1 cm) when the distance between the coils is fixed at *d* = *S*√3, accordingly to the results of Section 2.5. Figure 10a depicts the magnetic field profile plot along the *z*-axis, Figure 10b shows the field distribution in the *x*-*y* plane located half-way between the coils (*z* = √3/2 cm), and Figure 10c shows the field distribution in the *x*-*y* plane located half-way between the coils (*z* = √3/2 cm) for the quadrature SLC.

## 4. Discussion

The freely available simulator [7], based on the magnetostatic approach, can be useful for the first optimization of the unloaded RF coil, avoiding time-consuming and expensive approaches based on full-wave simulations. Moreover, as reported in the literature [22], such magnetostatic assumption is often verified at frequencies routinely used in clinical MRI applications, with the great advantage that reasonably accurate field calculation can be performed in a very short computation time (seconds), as compared to the full wave methods (hours) [23,24,25].

The present work, based on the previously validated code [7], proposes novel configurations, constituted by both axial field and transverse field surface coils.

Regarding the quadrature RF coil configurations proposed in the present paper, the magnetic field patterns are quite different for the three designs.

The SLC-SFC combination is characterized by high spatial inhomogeneity and a field maximum at some distance from the coil structure plane (Figure 6b), which can be useful when it is necessary to maximize the SNR at a given depth in the sample and, at the same time, minimize the signal from both the superficial layers (skin, fat) and the deep tissues (muscle). Moreover, even the magnetic field inhomogeneity in the *x*-*y* plane (Figure 5c,d) can be employed for achieving the highest SNR in specific hot spots of interest. 

Both the SLC-SBC (Figure 6a) and SFC-SBC (Figure 6c) configurations provide a high magnetic field amplitude in the central area near the coil structure plane and such behavior can be useful when high MR signals are required in close proximity to the sample surface. However, the two RF coil configurations showed different patterns in the *x*-*y* plane: SLC-SBC (Figure 5a,b) is like SLC-SFC but with a lower field intensity in the central area, and SFC-SBC (Figure 5e,f) shows a hot spot close to the *z* axis.

Regarding the proposed Helmholtz-like configurations, in Section 2.5 we presented analytical calculations for optimizing the magnetic field pattern along the coil’s axis, which were qualitatively verified for the SBC (Figure 8) and SFC (Figure 10) cases.

## 5. Conclusions

We presented different novel geometries for RF quadrature coils and used a magnetostatic approach [7] to simulate the B_1_ field. Novel quadrature surface coils constituted by a combination of SLC-SBC, SLC-SFC, and SFC-SBC were simulated and proposed for improving by a factor of up to √2 the SNR provided by a single surface coil. The optimal choice among the different configurations depends on the *B*_0_ orientation, as well as the envisaged application. For the first two configurations, the circularly polarized *B*_1_ field rotates in a plane orthogonal to the coils’ assembly and the penetration depth is large due to the presence of the SLC coil. With the last one, the *B*_1_ field rotates in a plane parallel to the coils’ assembly, and it is possible to reach a high RF field close to the coils but with a reduced penetration depth due to the rapid decrease in sensitivity of both the SFC and SBC. 

Moreover, the simulator was employed for studying three novel volume RF coil configurations with Helmholtz-like designs, and each structure is constituted by two identical surface coils (SLC-SLC, SFC-SFC, and SBC-SBC) separated by a given distance. We considered configurations generating linearly and circularly polarized RF fields. The Helmholtz-like configurations realized with transverse coils are new and have been studied from the point of view of the resulting field homogeneity. Such configurations can be useful for specific applications when the high sensitivity of a transverse coil is necessary but a slightly larger penetration depth, compared to the single coil case, is mandatory. 

We believe this work contains information useful for graduate students and researchers involved in the design of MRI/MRS RF coils.

## Data Availability

Available upon request to the authors.

## References

[1] Jin J. (1999). Electromagnetic Analysis and Design in Magnetic Resonance Imaging.

[2] Mispelter J., Lupu M., Briguet A. (2015). NMR Probeheads for Biophysical and Biomedical Experiments: Theoretical Principles & Practical Guidelines.

[3] Haase A., Odoj F., Von Kienlin M., Warnking J., Fidler F., Weisser A., Nittka M., Rommel E., Lanz T., Kalusche B. (2000). NMR probeheads for in vivo applications. Conc. Magn. Reson..

[4] Idiyatullin D., Corum C.A., Nixdorf D.R., Garwood M. (2014). Intraoral Approach for Imaging Teeth Using the Transverse B1 Field Components of an Occlusally Oriented Loop Coil. Magn. Reson. Med..

[5] Choi C.-H., Felder T., Felder J., Tellmann L., Hong S.-M., Wegener H.-P., Shah N.J., Ziemons K. (2020). Design, evaluation and comparison of endorectal coils for hybrid MR-PET imaging of the prostate. Phys. Med. Biol..

[6] Alfonsetti M., Sotgiu S., Alecci M. (2010). A Theoretical and Experimental Study on Transverse-Field Radio-Frequency Surface Coils. Measurement.

[7] Giovannetti G., Alecci M., Galante A. (2023). Biot–Savart-Based Design and Workbench Validation at 100 MHz of Transverse Field Surface RF Coils. Electronics.

[8] Foner S. (1996). The vibrating sample magnetometer: Experiences of a volunteer (invited). J. Appl. Phys..

[9] Sun K., Lai H., Lai Y., Liu C. (2019). Analysis of Coupler for Wireless Power Transmission. Proceedings of the 2019 International Conference on Precision Machining, Non-Traditional Machining and Intelligent Manufacturing (PNTIM 2019).

[10] Principe C., Giordano D., La Felice S., Giovannetti G., Devidze M. Measuring instruments and protocols in Archaeomagnetic dating: Magneto-stratigraphy in Archaeology and Volcanology. Proceedings of the 2019 IMEKO TC-4 International Conference on Metrology for Archaeology and Cultural Heritage.

[11] Letcher J.H. (1989). Computer-assisted design of surface coils used in magnetic resonance imaging. I. The calculation of the magnetic field. Magn. Reson. Imag..

[12] Hoult D.I. (2000). The principle of reciprocity in signal strength calculations? A mathematical guide. Conc. Magn. Reson..

[13] Chen C.-N., Hoult D., Sank V. (1983). Quadrature detection coils—A further √2 improvement in sensitivity. J. Magn. Reson..

[14] Hyde J.S., Jesmanowicz A., Grist T.M., Froncisz W., Kneeland J.B. (1987). Quadrature Detection Surface Coil. Magn. Reson. Med..

[15] Versluis M.J., Tsekos N., Smith N.B., Webba A.G. (2009). Simple RF design for human functional and morphological cardiac imaging at 7 tesla. J. Magn. Reson..

[16] Giovannetti G., Frijia F., Hartwig V., Attanasio S., Menichetti L., Vanello N., Positano V., Ardenkjaer-Larsen J.H., Lionetti V., Aquaro G.D. (2013). Design of a quadrature surface coil for hyperpolarized 13C MRS cardiac metabolism studies in pigs. Conc. Magn. Reson. Part B Magn. Reson. Eng..

[17] Giovannetti G., Frijia F., Flori A., Montanaro D. (2019). Design and Simulation of a Helmholtz Coil for Magnetic Resonance Imaging and Spectroscopy Experiments with a 3T MR Clinical Scanner. Appl. Magn. Reson..

[18] Bin M., Kai-Wen H., Wei-Min W. (2010). A novel radio frequency coil for veterinary magnetic resonance imaging system. Chin. Phys. B.

[19] De Zanche N., Massner J.A., Leussler C., Pruessmann K.P. (2008). Modular design of receiver coil arrays. NMR Biomed..

[20] Vaughan J.T., Griffiths J.R. (2012). RF Coils for MRI.

[21] Ginsberg D.M., Melchner M.J. (1970). Optimum Geometry of Saddle Shaped Coils for Generating a Uniform Magnetic Field. Rev. Sci. Instrum..

[22] De Pellegars P., Pan L., Sidi-Boulenouar R., Nativel E., Zanca M., Alibert E., Rousset S., Cardoso M., Verdeil J.-L., Bertin N. (2020). Homogenous nuclear magnetic resonance probe using the space harmonics suppression method. J. Sens. Sens. Syst..

[23] Hartwig V., Vanello N., Giovannetti G., De Marchi D., Lombardi M., Landini L., Santarelli M.F. (2011). B1+/actual flip angle and reception sensitivity mapping methods: Simulation and comparison. Magn. Reson. Imag..

[24] Hartwig V., Giovannetti G., Vanello N., Landini L., Santarelli M.F. (2010). Numerical Calculation of Peak-to-Average Specific Absorption Rate on Different Human Thorax Models for Magnetic Resonance Safety Considerations. Appl. Magn. Reson..

[25] Giovannetti G., Hartwig V., Viti V., Zadaricchio P., Meini L., Landini L., Benassi A. (2008). Low field elliptical MR coil array designed by FDTD. Conc. Magn. Reson. Part B Magn. Reson. Eng..

